# Insights into human TLR9 structural dynamics and recognition of pathogen-associated CpG DNA using molecular dynamics simulation

**DOI:** 10.1016/j.isci.2026.117060

**Published:** 2026-08-12

**Authors:** Santanu Sasidharan, Vijayakumar Gosu, Donghyun Shin

**Affiliations:** 1 Michael Smith Laboratories, The University of British Columbia, Vancouver, BC, Canada; 2 Department of Physics and Astronomy, The University of British Columbia, Vancouver, BC, Canada; 3 Department of Animal Biotechnology, Jeonbuk National University, Jeonju 54896, Republic of Korea; 4 Department of Agricultural Convergence Technology, Jeonbuk National University, Jeonju 54896, Republic of Korea

**Keywords:** toll-like receptor 9, CpG ssDNA, infectious diseases

## Abstract

Human toll-like receptor 9 (TLR9) is a key pattern-recognition receptor that detects unmethylated CpG motifs in pathogen single-stranded (ss) DNA. Although structural information for non-human TLR9 ectodomains exists, the conformational dynamics of human TLR9 remain poorly understood. Here, we modeled the TLR9 ectodomain and performed all-atom simulations of apo TLR9, CpG-bound TLR9, and CpG-immunomodulator (IM) co-bound TLR9. We show that the apo ectodomain formed a stable pre-assembled homodimer and CpG ssDNA was observed to bind with a 2:2 stoichiometry, bridging both monomers and inducing localized rearrangements within the leucine-rich repeat region. CpG ssDNA binding enhanced Z-loop flexibility by ∼2 folds in the TLR9_CpG and TLR9_CpG_IM complexes. Furthermore, CpG ssDNA and IM engagement reorganized inter-monomer contacts and revealed previously unrecognized functional residues like H612, H641, T642, and K690, that supports cooperative ligand stabilization. Together, these simulations identify candidate structural transitions and putative allosteric nodes in human TLR9 for future experimental studies.

## Highlights

• The ectodomain of TLR9 was simulated in its apo form and in complex with CpG DNA and IM
• Apo-TLR9 simulations suggest that TLR9 exists as a preformed dimer at the ectodomain
• Binding of CpG DNA to TLR9 exposes the IM binding site located near LRR18-19
• Interaction analysis predicted functional important residues: H612, H641, T642, and K690

## Graphical abstract

**Figure:**
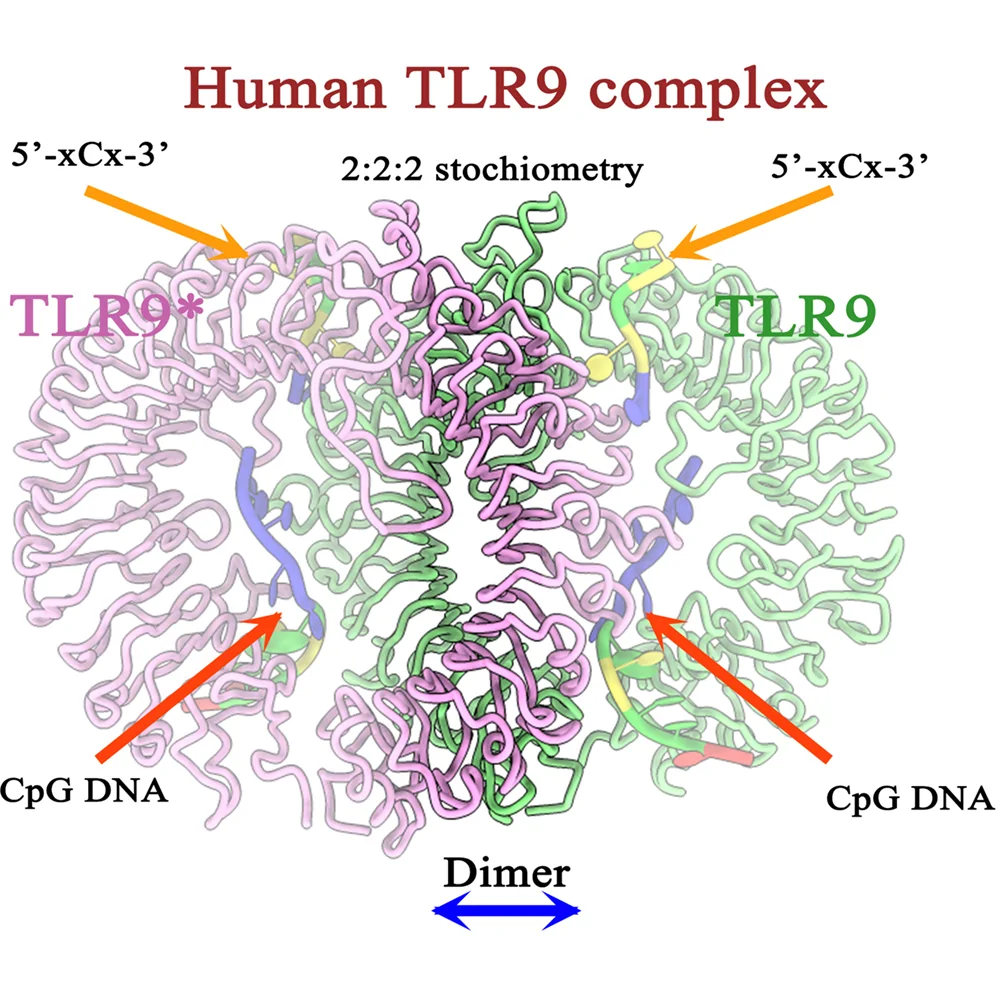

## Teaser

Structural biology; Biophysics; Bioinformatics

## Introduction

Human immune responses are mediated by innate and adaptive immunity. Innate immunity is the first line of defense against pathogens, whereas adaptive immunity raises pathogen-specific responses. Cells that confer innate immunity to humans can detect various pathogens using pattern recognition receptors (PRRs).^1^ PRRs are a class of receptors that recognize specific molecular patterns in pathogens, such as pathogen-associated molecular patterns (PAMPs) and trigger their respective immune responses.^2^ Most characterized PAMPs are nucleic acids of pathogens, such as single-stranded (ss) and double-stranded (ds) DNA and RNA. These PAMPs activate PRRs to produce type-1 interferons and proinflammatory cytokines to combat pathogens. In humans, only a few well-known PRRs are present which includes toll-like receptors (TLRs),^3^ nucleotide-binding oligomerization domain-like receptors^4^ and retinoic acid-inducible gene I-like receptors.^5^ It is important that PRRs precisely recognize nucleic acids from pathogens and avoid self-recognition of host nucleic acids. This precise recognition is achieved by regulation, such as localization and processing of pathogen nucleic acids, post-translational processing of PRRs, and specific interactions of PRRs with nucleic acids.^2^ These PRR regulations are crucial for understanding the mechanisms of the immune response, autoimmune diseases, as well as the development of vaccines and other therapeutics.^1^

One of the well-known PRR members is the TLRs. TLRs are transmembrane proteins that are evolutionarily conserved between insects and vertebrates.^3^ These receptors are essential for the innate immune system as they recognize PAMPs, such as lipopolysaccharides, peptidoglycans, flagellin, and unmethylated cytosine-phosphate-guanine (CpG) DNA.^6^ The activation of TLRs leads to a signaling pathway involving the transcription factors NF-kB and AP-1. These transcription factors work synergistically to initiate cytokines, chemokines and matrix-degrading enzyme for bacterial and viral infections. There are 10 TLRs recorded in humans,^7^ 13 in mice^8^ and at least 6 (TLRs 2–4 and 7–(9) in horses.^9^^,^^10^^,^^11^ Among TLRs, human TLR3, TLR7/TLR8, TLR9 and mouse TLR13 recognize nucleic acids and are localized in the endosomal compartment. TLR3, TLR7/TLR8 and TLR9 sense dsRNA, ssRNA and CpG DNA respectively^12^^,^^13^ while, mouse TLR13 binds to 23 ribosomal RNA.^14^^,^^15^ TLR7 and TLR8 can recognize ssRNAs and TLR9 is specific to single-stranded DNA (ssDNA) with a CpG dideoxynucleotide motif. Since TLR7, TLR8, and TLR9 recognize single-stranded nucleic acids, they are grouped under the TLR7 subfamily. These TLRs have the same number of leucine-rich repeats (LRRs).

**Figure 1:**
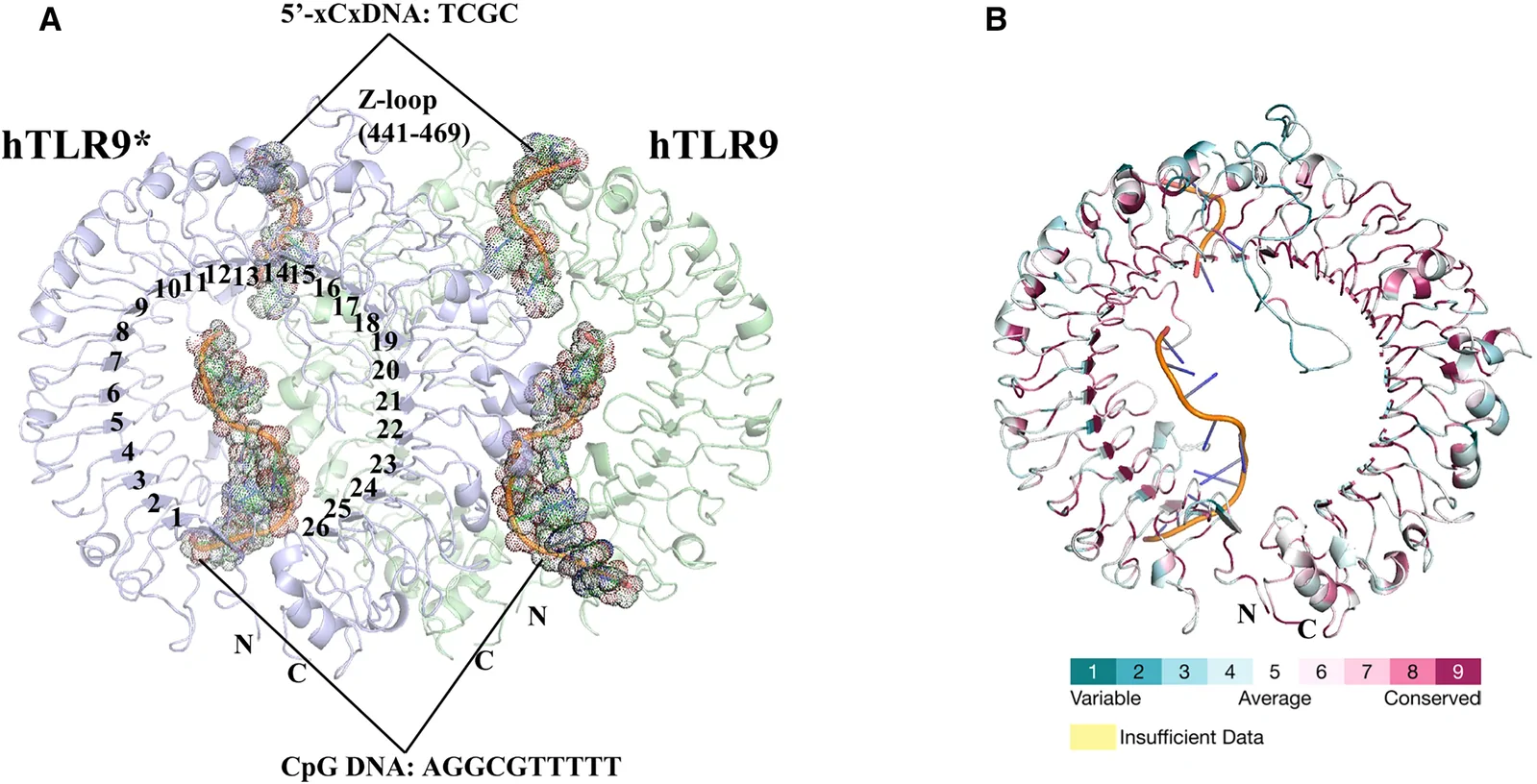
TLR9 complex Structure of CpGDNA- and IM-bound TLR9 complex (A). Evolutionarily conserved regions of TLR9 (B).

As mentioned earlier, TLR9 in the TLR7 subfamily recognizes unmethylated CpG motifs in ssDNA of bacteria and viruses and is initially localized in the endoplasmic reticulum (ER) before exiting to the endosomal compartments.^16^ The ectodomain of TLR9 is oriented toward the lumen of endosomes, while its C-terminal domain extends into the cytoplasm. Multiple mechanisms maintain the proper localization of TLRs, including motifs in the cytoplasmic tail that retain TLR9 in the ER and TLR9 phosphorylation.^17^ TLR9 also contains LRRs i.e., LRR1–26^18^ and between LRR14 and LRR15 lies a long loop region of 40–50 amino acids (aa) called the Z loop. Several studies have shown that the Z-loop cleavage is essential for TLR7 subfamily activation (Figure 1A).^18^ Structurally, TLR9 comprises an N-terminal LRR (26–61 aa), LRR1–14 (62–440 aa), Z loop (441–469), LRR15–26 (470–772 aa), C-terminal LRR (773–818 aa), a transmembrane segment (819–839 aa), and a cytoplasmic toll/interleukin-1 receptor/resistance protein (TIR) domain (840–1,032 aa).^19^ Cleaved TLR9 contains 471–1,032 aa, spanning the LRRs (15–26) to the C-terminal end.^20^ TLR7 and TLR8 simultaneously bind two ligands: a ssRNA and a mononucleoside for dimerization.^21^^,^^22^^,^^23^ On the other hand, The TLR9 dimer harbors two ligand binding sites: one for the CpG ssDNA and the other for the 5′-xCx DNA. TLR9 dimerization is enhanced by 5′-xCx DNA in the presence of CpG DNA, consistent with synergistic ligand engagement.^24^^,^^25^ Crystal structures of *Equus caballus* TLR9 show that two receptor ectodomains bind two DNA ligands to form the active complex, and this architecture is highly relevant to human TLR9 given the >80% sequence conservation in the ligand-binding and dimerization regions ligands.^24^^,^^25^ Mouse TLR9 structures likewise reveal a dimer engaging two DNA molecules, indicating that this stoichiometry is conserved across mammals.^21^ Functional studies in human TLR9 further demonstrate that TLR9 requires two CpG motifs, consistent with a bivalent ligand-receptor arrangement.^26^^,^^27^^,^^28^^,^^29^ Pohar et al. also showed that the short ssDNA containing a 5′-TCG motif augments CpG-containing DNA-induced TLR9 structural rearrangement.^28^

Multiple studies have been conducted on mouse, bovine, and horse TLR9; however, little is known about dynamics and key interactions between human TLR9, CpG ssDNA, and IM complex.^10^^,^^21^^,^^30^ The crystal structure of human TLR9 has not yet been elucidated but the advancement in computational tools now allows us to model a protein of interest using homologous structures. In this study, we modeled human TLR9 based on the crystal structure of *Equus caballus* TLR9 (Figure 1A) and simulated TLR9 in complex with CpG ssDNA and a 5′-xCx DNA immunomodulator (IM) to understand the dynamic conformational changes in TLR9 and the mechanism of ssDNA ligand engagement. We report various intra-, inter-monomer, and also TLR9-CpG-IM interactions that might help design ligands for therapeutic purposes.

## Results

### Dynamics of apo- and CpG ssDNA and IM-bound TLR9 dimer complexes

**Figure 2:**
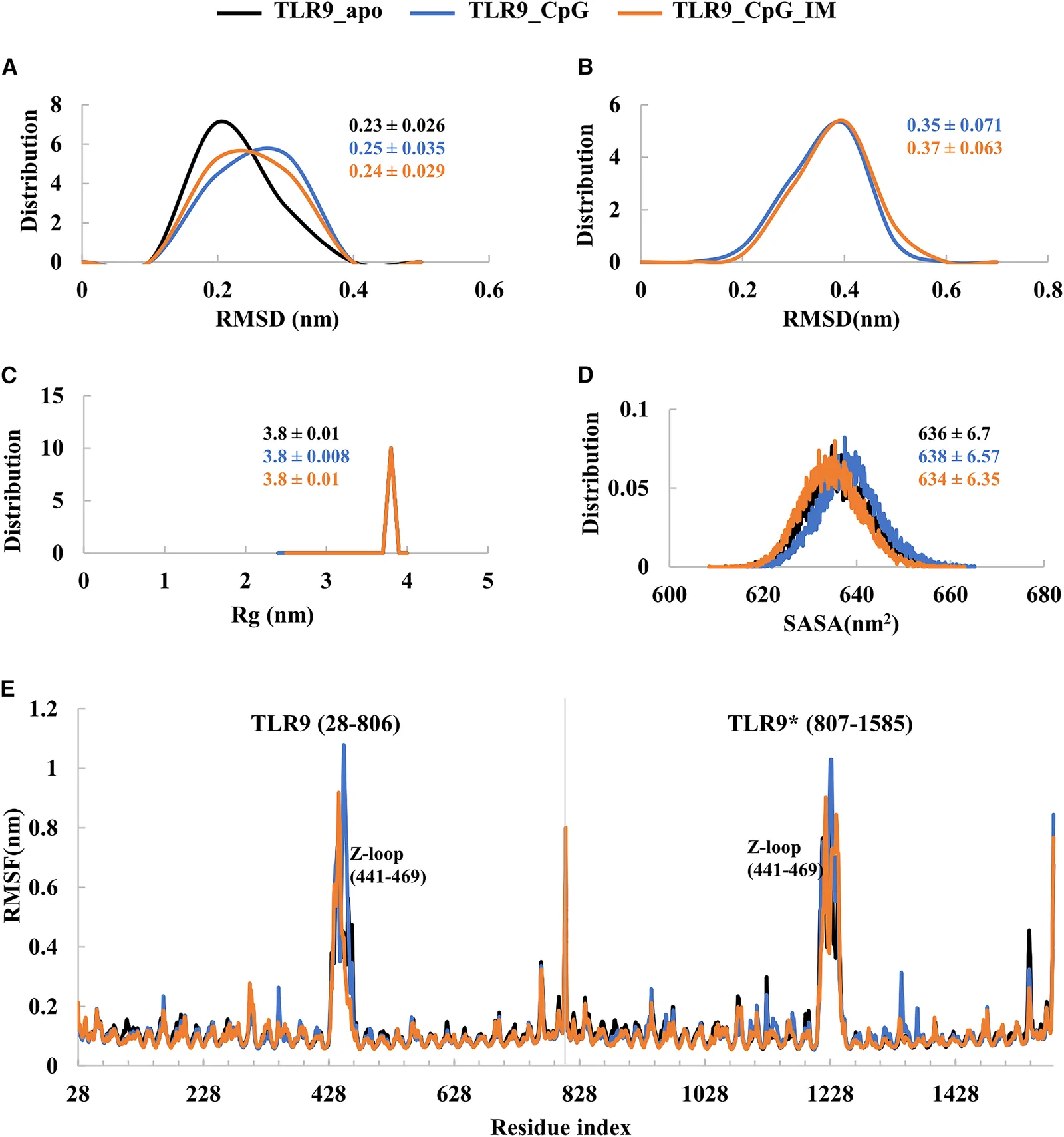
Structural dynamics of TLR9 complexes Density distributions of RMSD for TLR9 backbone (A) and both (ssDNA and IM) ligands (B). Density distribution of radius of gyration (Rg) for TLR9 backbone (C). Density distribution of solvent-accessible surface area (SASA) for TLR9 (D). RMSF of TLR9 backbone (E). The average values along with standard deviation are also given.

Initial sequence homology characterization with other TLR9 ECDs, mapped onto the modeled human TLR9 structure, is shown in Figure 1B. The modeled TLR9_apo had an root-mean-square deviation (RMSD) value of 0.55 Å with mouse TLR9 and 0.45 Å with equine TLR9. The figure demonstrates the conservation of ligand-exposed and ligand-unexposed regions of the LRRs. We observed 26 LRR repeats in the human TLR9 model, similar to mouse and horse TLR9 structures previously observed.^21^^,^^25^ We then, prepared three different system, i.e., TLR9_apo, TLR9_CpG, and TLR9_CpG_IM and ran three independent all-atom simulations of 300 ns each (totaling to 900 ns for each system). Post simulation, the protein backbone RMSD was calculated to assess stability of the protein in the simulations. The backbone RMSD distribution of the three complexes show small deviations and unimodal distribution with average values of 0.23 ± 0.026, 0.25 ± 0.035, and 0.24 ± 0.029 nm for TLR9_apo, TLR9_CpG, and TLR9_CpG_IM, respectively, suggesting stability of the simulated complexes (Figure 2A). The stable deviation in TLR9_apo during the simulations suggests that TLR9 exists as a preformed dimer, at least in the ectodomain (ECD). This is consistent with a previous study showing that human TLR9 exists as a preformed dimer.^31^ The RMSD distributions of the ligand CpG in TLR9_CpG and of both ligands, CpG and IM, in TLR9_CpG_IM complexes were clustered at 0.35 ± 0.07 and 0.37 ± 0.06 nm, respectively. The minimal deviation in the ligand RMSD indicated stability and possible interactions with the TLR9 homodimer (Figure 2B).

The radius of gyration (Rg) of the simulated complexes was assessed to evaluate the rigidity and compactness of the protein complexes during the simulation period. The average Rg values of the TLR9_apo, TLR9_CpG, and TLR9_CPG_IM were 3.8 ± 0.01, 3.8 ± 0.008, and 3.8 ± 0.01 nm, respectively. The Rg of TLR9_apo, TLR9_CpG, and TLR9_CpG_IM complexes showed a unimodal distribution and had a broader distribution of Rg values (Figure 2C). We also calculated the solvent accessible surface area (SASA) to determine any major folding or conformational changes. The average SASA value was 636 ± 6.7, 638 ± 6.57, and 634 ± 6.35 nm for TLR9_apo, TLR9_CpG, and TLR9_CpG_IM, respectively, indicating no major conformational events (Figure 2D). Further, the per-residue fluctuations of the protein backbone were calculated with the root-mean-square fluctuation (RMSF) tool. The RMSFs of all three complexes were similar, except for parts of the Z loop (441–469 aa) region as well as neighboring residues of Z loop of each TLR9 monomer (Figure 2E). Notably, the Z-loop fluctuation was approximately 2-fold higher in the TLR9_CpG and TLR9_CpG_IM complexes than in TLR9_apo. Z-loop dynamics are crucial for binding and adapting CpG and IM to TLR9,^32^ and our results support this hypothesis. In addition, we observed similar per-residue fluctuations for both the monomers of TLR9, demonstrating a positive correlation between the dynamics of the monomers (Figure 2E). The simulations show that all TLR9 complexes remain structurally stable, with ligand binding primarily enhancing Z-loop flexibility, supporting its central role in accommodating CpG and IM while preserving the integrity of the preformed TLR9 dimer.

### Hydrogen bonds and interaction energy of apo- and CpG ssDNA and IM-bound TLR9 dimer complexes

**Figure 3:**
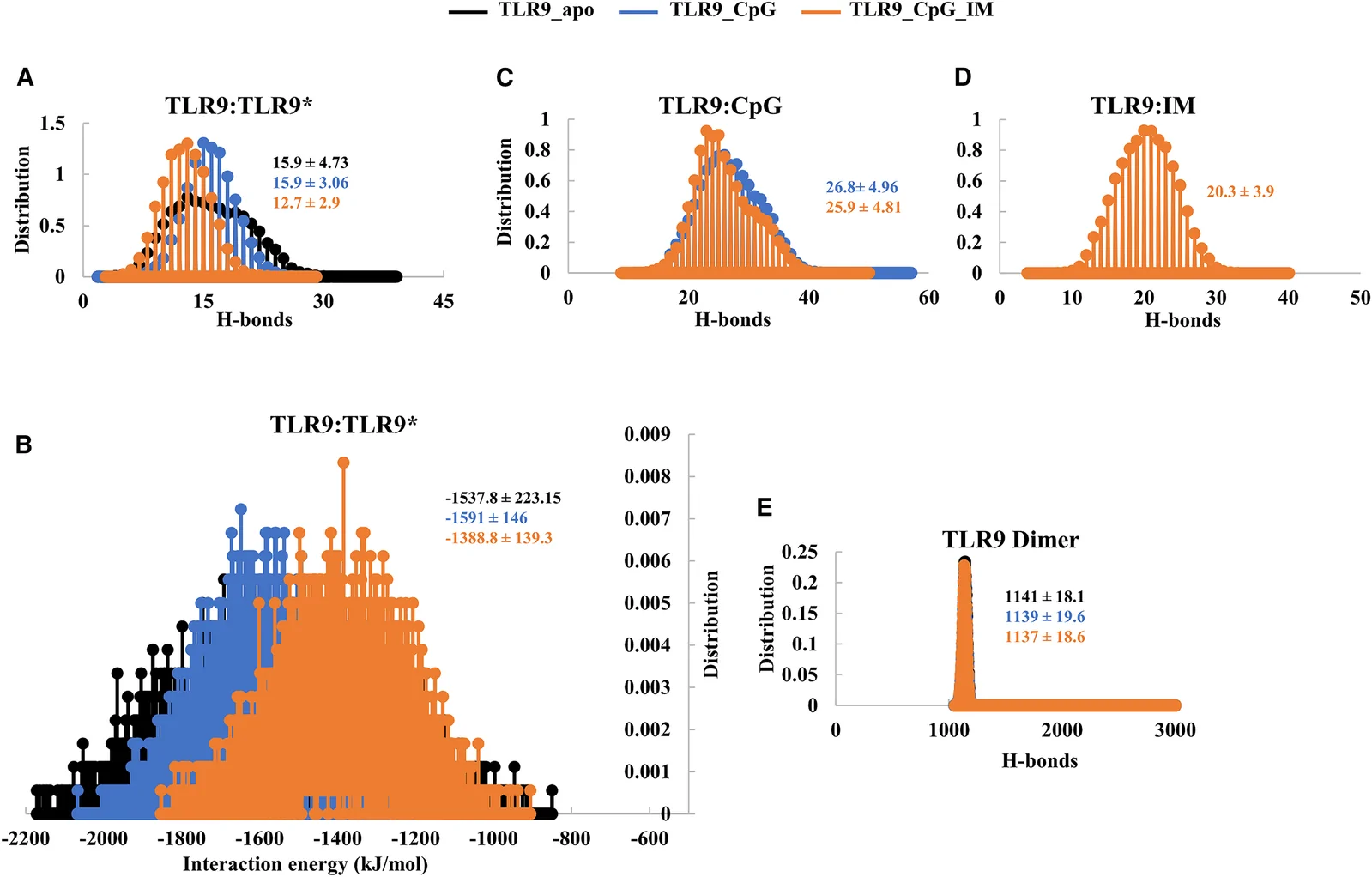
H-bond analysis The distribution of H bonds (A) and interaction energy (B) between TLR9 and TLR9∗. The distribution of H bonds between TLR9 and CpG ssDNA (2:2 stoichiometry) (C), between TLR9 and IM (2:2 stoichiometry) (D). Distribution of intra-H bonds within the TLR9 dimer (E). The average values along with standard deviation are also given.

Furthermore, to check the intra- and inter-molecular hydrogen bonds within TLR9 complexes, we analyzed hydrogen bonds using a cutoff distance 0.35 nm and an angle of 30° between TLR9 monomers, as well as between TLR9 and CpG. The average hydrogen bonds and interaction energy observed between TLR9 monomers (TLR9 and TLR9∗) in TLR9_apo, TLR9_CpG, and TLR9_CPG_IM were 16 ± 5 and −1,537 ± 223 kJ/mol, 16 ± 4 and −1,591 ± 146 kJ/mol, and 13 ± 3 and −1,388 ± 139 kJ/mol, respectively (Figures 3A and 3B). From the results, the hydrogen bonds demonstrate the intactness of the TLR9 dimer in all simulated protein complexes. The distribution of H-bonds was broader for TLR_apo which reduced in TLR_CpG and TLR9_CpG_IM complexes. This heterogeneity in interaction may be a result of CpG and IM binding that reorganizes the dimer interface interactions. The interaction energy further suggests the stabilization of TLR9 dimer upon CpG binding. Although the hydrogen bonds remained similar in TLR9_apo and TLR9_CpG, there was a modest increase in their interaction energies which could be as a result of structural stabilization of TLR9 monomers upon CpG motif binding. By contrast, TLR9_CpG_IM had significantly fewer inter-molecular hydrogen bonds and reduced interaction energy between its monomers, indicating the reallocation of H-bonds between TLR9-TLR9∗ dimer to IM that binds in the dimer interface (Figure 3A).

The average number of hydrogen bonds between CpG and TLR9 (2:2) in TLR9_CpG and TLR9_CpG_IM complex was 27 ± 5 and 26 ± 6, respectively (Figure 3C). There was no increase in hydrogen bonds between CpG and TLR9 in TLR9_CpG_IM complex and TLR9_CpG complex which suggests that the binding of IM does not affect CpG binding to TLR9. In addition, the hydrogen bonds between TLR9 and IM from the TLR9_CpG_IM complex was around 20, indicating the strong interaction of IM with individual TLR9 monomers (Figure 3D). The intra-molecular hydrogen bonds within each TLR9 monomer did not vary much among the three complexes TLR9_apo, TLR9_CpG, and TLR9_CpG_IM (Figure 3E).

We analyzed the H-bond occupancy (in %) between TLR9 and TLR9∗ (∗ are residues in TLR9∗, hereafter) over 300 ns and observed H-bond between residues Q797∗-A736 (59%), R348∗-G565 (58%), Q797-A736∗ (53%), and H260-Q562∗ (52%) in TLR9_apo complex. For TLR9_CpG, we calculated Y180-E616∗ (70%), Q797-A736∗ (55%), Q797∗-A736 (54%), R348∗-G565 (49%), and K738∗-G791 (49%). The IM bound complex TLR9_CpG_IM showed Y180-E616∗ (66%), Q797-A796∗ (60%), Y180∗-E616 (60%), and H260-G563∗ (48%). We note that Y180-E616∗ had a maximum of 18% occupancy in TLR9_apo, while this pair in TLR9_CpG and TLR9_CpG_IM show more than 60% H-bond occupancy. Collectively, these results suggest that CpG and IM binding induce rearrangements within the ectodomain that may represent early structural events preceding downstream signaling, although our simulations cannot directly capture TIR-domain dynamics.

### Principal components, free energy landscape, and dynamic cross-correlation matrix analysis

**Figure 4:**
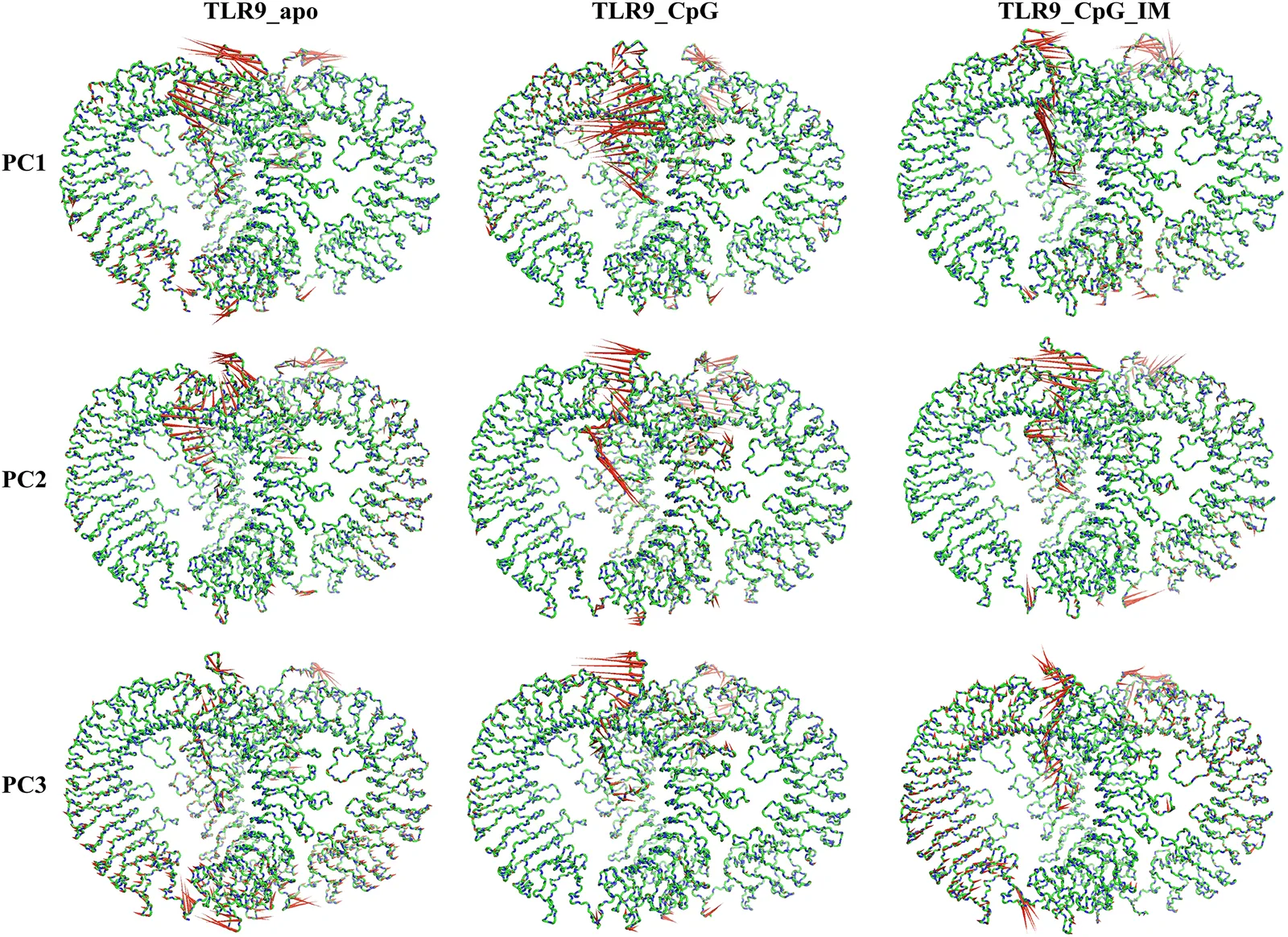
Conformational changes during simulations Porcupine plots depicting the regions of the dominant motions from the top principal components (PC1, PC2, and PC3). Orange arrow vectors indicate the direction and relative magnitude of residue displacements, highlighting the dominant collective motion predicted by PCA.

Following stability validation, we assessed variations in the global motions of TLR9 complexes, particularly those induced by CpG and IM binding. To this end, the principal components (PCs) of the concatenated trajectory using protein backbone atoms were analyzed. The top three PCs contributed substantially to the collective motions of all systems, accounting for ∼50% of the total variance (Figure S1). Phase-space projections (PC1 vs. PC2, PC2 vs. PC3, and PC1 vs. PC3) revealed periodic transitions separated by minimal energy barriers. Because the top PCs exhibited the largest amplitudes, porcupine plots were generated to visualize the directionality of motion (Figure 4). Across all complexes, the Z loop displayed the most pronounced conformational fluctuations, whereas the remainder of the ectodomain exhibited comparatively modest motions, consistent with the RMSF profiles described earlier (Figure 2E).

**Figure 5:**
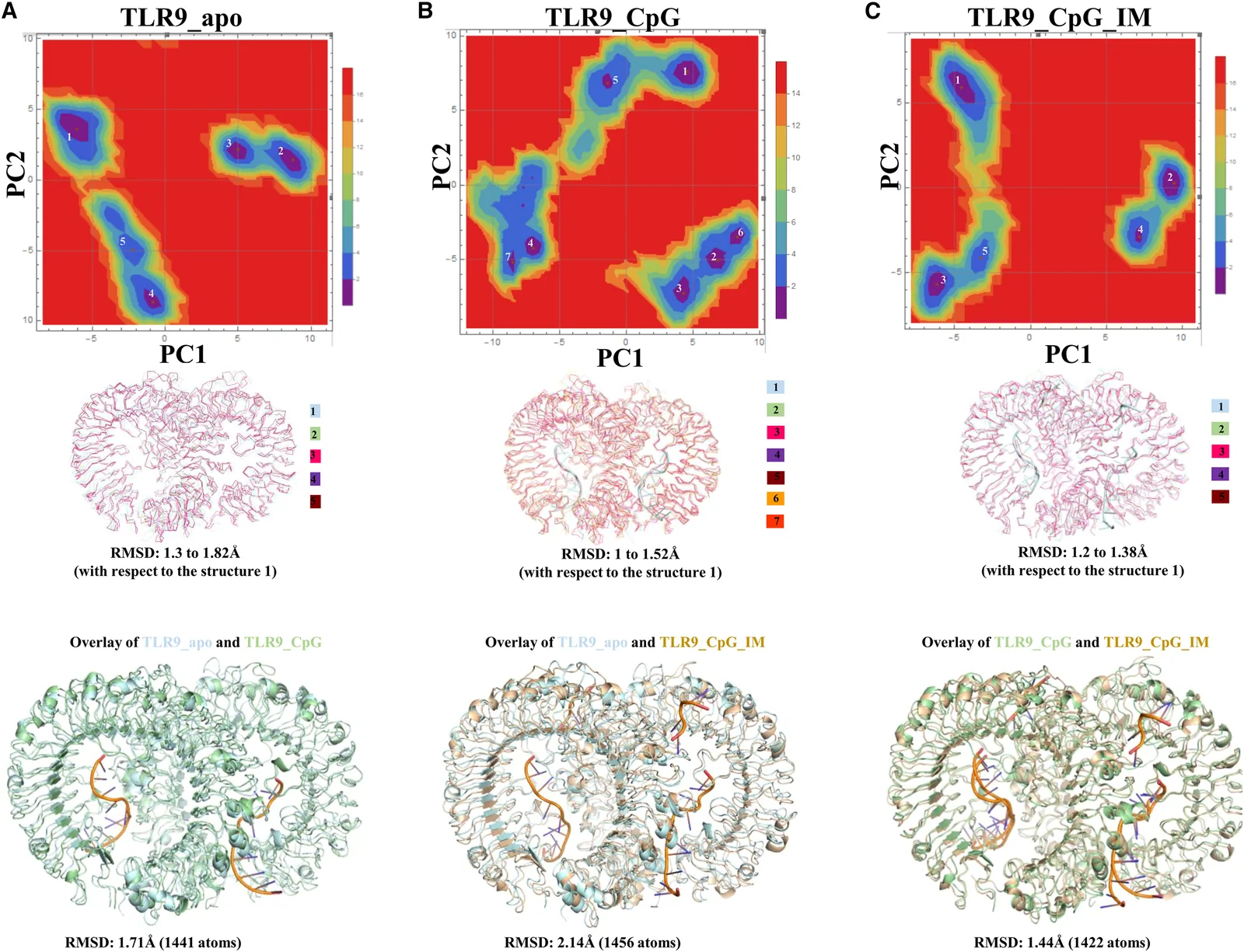
Free-energy landscapes FEL plots for TLR9_apo (A), TLR9_CpG (B), and TLR9_CpG_IM (C) using PC1 and PC2 coordinates. Corresponding structures from the metastable states are superimposed (middle). Representative structures from TLR9_apo, TLR9_CpG, and TLR9_CpG_IM are overlaid to show regions with variance.

**Figure 6:**
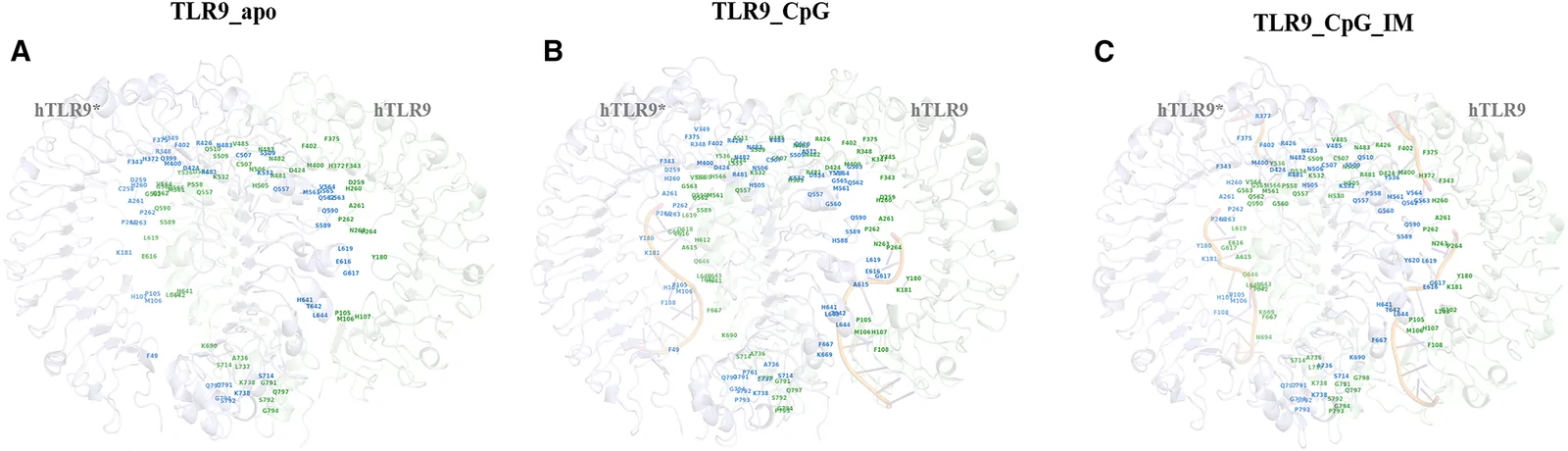
TLR9 Dimer interface The residues considered within 4 Å of each TLR9 monomer at the dimer interface TLR9_apo (A), TLR9_CpG (B), TLR9_CpG_IM (C) are shown.

We computed the free energy landscape (FEL) of the three simulated complexes to identify the meta-stable states of these ligand complexed proteins and characterize conformational heterogeneity. The FELs in Figure 5 show five, seven, and five low-energy populated states for TLR9_apo (Figure 5A), TLR9_CpG (Figure 5B), and TLR9_CpG_IM (Figure 5C), respectively. Visual inspection indicated that most free-energy differences originated from loop regions, which constitute a substantial portion of the receptor. The dimer interface was predominantly lined with hydrophilic and charged residues, along with a smaller number of hydrophobic (Leu) residues, which formed hydrogen bonds with backbone carbonyls of asparagine, histidine, and alanine (Figure 6; Figure S2). The increased number of metastable states in the TLR9_CpG complex likely reflects subtle conformational adjustments upon CpG binding, whereas the reduced number of states in the TLR9_CpG_IM complex suggests that IM binding stabilizes the CpG-bound dimer.

**Figure 7:**
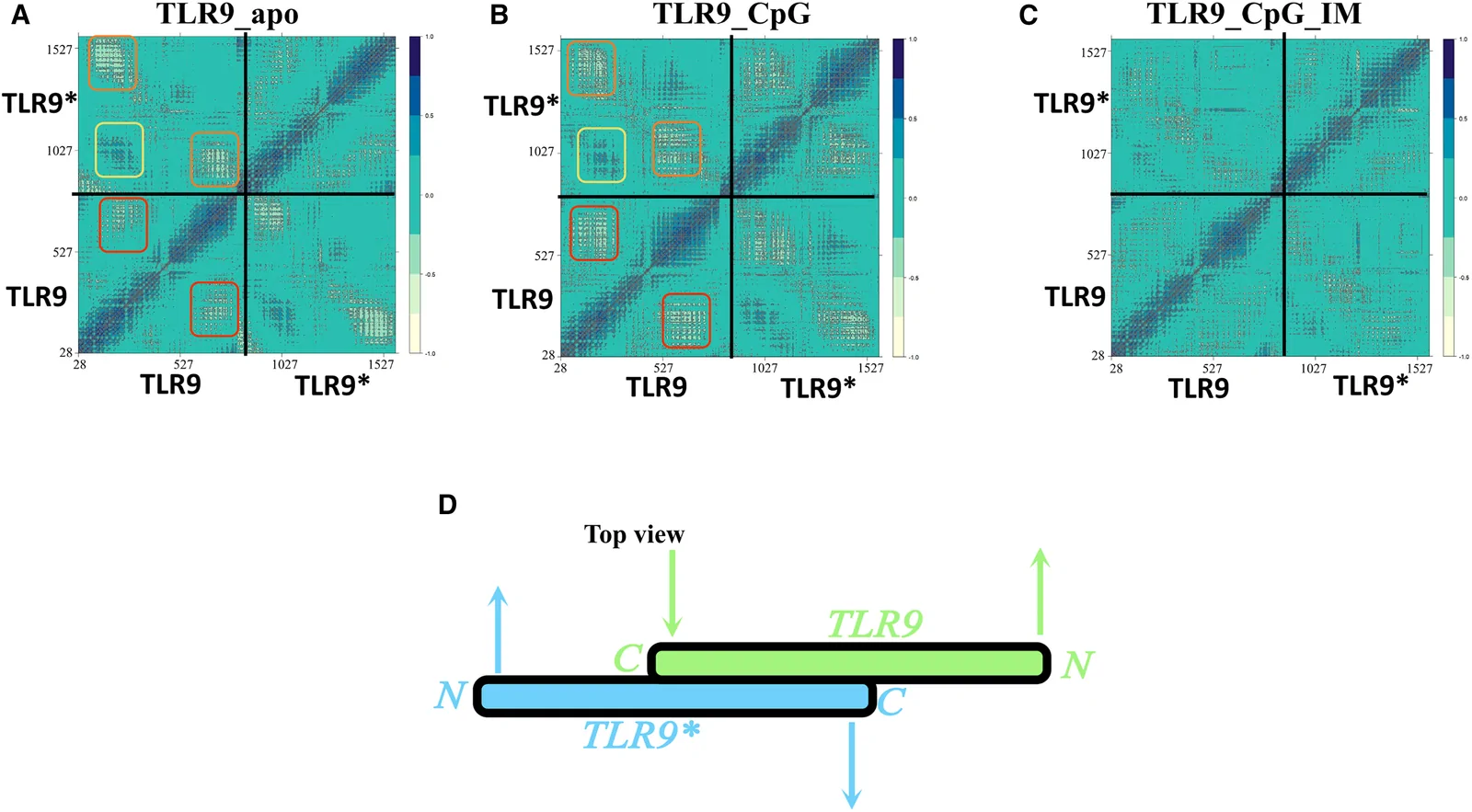
Dynamic cross-correlation matrix of all three systems TLR9_apo (A), TLR9_CpG (B), and TLR9_CpG_IM (C). Map shows that the N terminus of TLR9 moves anti-parallel to the C terminus of TLR9 (red boxes) and even more strongly to the C terminus of TLR9∗ (orange boxes). The N terminuses of TLR9 and TLR9∗ display positively correlated motion (yellow box), indicating movement in the same direction. Schematic representation showing the possible monomer motion and how the DCCM relationships hints on a hinge-like interaction interface between the central regions of TLR9 and TLR9∗ (D).

We further analyzed the dynamic cross-correlation matrix (DCCM) plots of the three structures to determine the correlated motion of TLR9. The DCCM plots for TLR9_apo demonstrated a weak anticorrelation between the 100–300 aa region and 700–800 aa region of TLR9 monomers and a stronger anti-correlation between the 100–300 aa region of TLR9 and 700–800 aa region of TLR9∗ (Figure 7A). The anti-correlation suggests that N terminus of TLR9 moves in an anti-parallel fashion, weakly to C terminus of TLR9 (see red boxes in Figure 7A) and strongly to the C terminus of TLR9∗ (see orange boxes in Figure 7A). The anti-correlation is stronger at the C terminus TLR9∗ because of the cumulative motion of N terminus TLR9 and TLR9∗. There is positive correlation between the N terminuses of TLR9 and TLR9∗ which suggest that they move in the same vector direction (see yellow box in Figure 7A). These correlations are consistent with the preformed dimer arrangement reported previously and reflect ectodomain-level motions observed in our simulations.^31^ The results are also in line with the PCA analysis where the above-mentioned regions had opposite global motions in TLR9_apo and TLR9_CpG (Figure 4).

In contrast, for TLR9_CpG, the anti-correlation observed earlier, between the 100–300 aa and 700–800 aa regions of individual monomers increased upon CpG binding while the anti-correlation with 700–800 aa of TLR9∗ reduced (Figure 7B). The N terminus positive correlation at TLR9 and TLR9∗ was observed in TLR9_CpG complex too. We also observed that the region downstream of Z loop was strongly correlated within the monomer after CpG binding. As expected, these correlations in complexes (TLR9_apo, TLR9_CpG_IM) were not observed for TLR9_CpG_IM complex, which might be as a result of relaxed dimer conformations upon IM binding (Figure 7C). The global motions observed in the PCA analysis corroborated the DCCM results and schematic view of the correlation/anticorrelation motions observed from DCCM were shown on Figure 7D. We also calculated the distance between the A765-A765∗ (C-ter LRR26) residues of the TLR9-TLR9∗ complex to analyze if the monomers moved away from each other at the C-terminal transmembrane domain. We did not observe any large changes, implying a stable dimer and TLR9_apo had heterogeneous distribution, followed by TLR9_CpG and then TLR9_CpG_IM (Figure S3). However, the decreasing trend in distance distribution and the decrease in heterogeneity might be a result of the monomers moving toward each other when CpG and IM bind to the dimer. Overall, CpG and IM binding reshape TLR9 dynamics by concentrating global motions in the Z loop, reorganizing long-range correlated movements, and stabilizing the dimer into fewer, more compact conformational states without disrupting its overall integrity.

### Molecular interaction between TLR9 and TLR9∗, CpG DNA, and IM in metastable complex states

We further picked representative metastable states from each FEL cluster identified in the previous section and analyzed the inter-monomeric (TLR9-TLR9∗) and ligand protein interactions. These interactions provide critical insight into how ligand binding reshapes TLR9 dynamics. Our simulations revealed several residues not previously implicated in human TLR9 activation, including H612, H641, T642, and K690. In the apo state, inter-monomer contacts such as P105–H641∗/T642∗, H641–P105∗, T642–P105∗, and D618/H107-H107∗/D618∗ were observed in addition to hydrogen-bond interactions.

**Figure 8:**
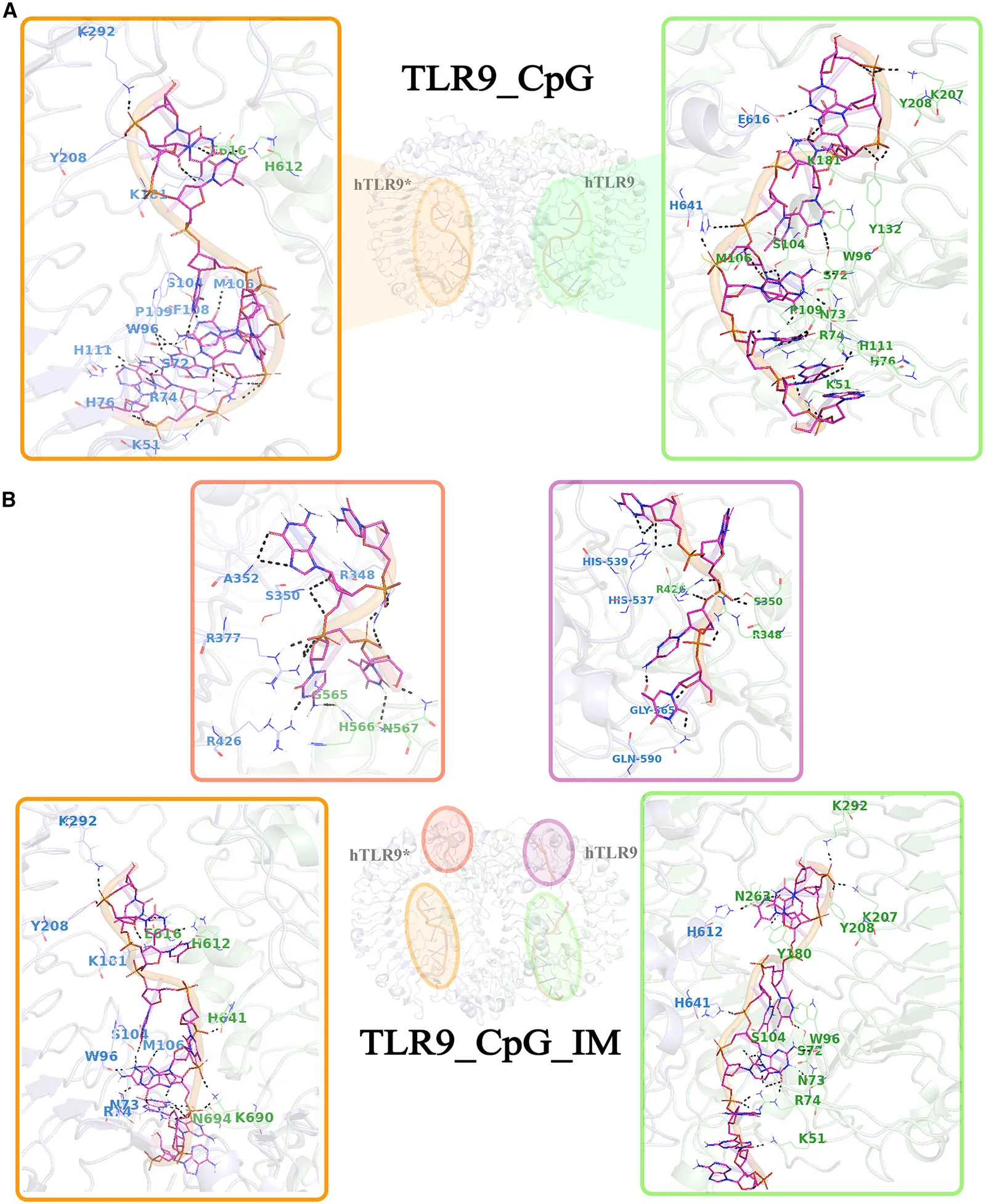
CpG DNA and IM binding site residues of TLR9 The residues considered within 4 Å of CpG DNA complex (A), and CpG and IM (B) binding site of TLR9. The single representative low energy complex was considered for clarity from FEL, hence, some of the residues discussed in the main text may not be present in the figure.

We then examined states 1, 2, 4, and 5 as representatives of the four FEL clusters in TLR9_CpG complex (Figure 8A). The 10 bp ssDNA was stabilized by conventional hydrogen bonds, electrostatic interactions, and hydrophobic contacts. Residues S104, P105, and M106 in LRR2 formed hydrogen bonds, while F108 formed a pi-pi T-shaped interaction with 4C/4C∗ of the ssDNA across all states. In addition, R74 and H76 in LRR1 formed electrostatic interactions with 3G/3G∗ and 4C/4C∗ of the ssDNA, and W47 (N-terminal LRR) together with W96 (LRR2) established pi-pi stacking interactions with 6 T/6 T∗ and 7 T/7 T∗ of the ssDNA. K181 (LRR5), K207 (LRR6), and K292 (LRR9) interacted electrostatically with 10 T/10 T∗, whereas Y208 and E616 (LRR20) formed hydrogen bonds with 10 T of the ssDNA. In clusters 1, 4, and 5, K181 and K207 also stabilized 9 T of the ssDNA. In the apo form, K181 (24% H-bond occupancy)/Y180 (18% H-bond occupancy) interacted with E616∗ but upon ligand binding, K181 shifted to form hydrogen bond with the CpG ssDNA while Y180-E616 H-bond occupancy increased to 70%.

Surprisingly, K690 (LRR23) of TLR9 engaged the CpG ssDNA ligand bound to TLR9∗ electrostatically. Whereas H612 (LRR20), H641, and T642 (LRR21) formed hydrogen bonds with 5G, 6T, 8T, and 9T of the CpG ssDNA bound to TLR9∗. These findings suggest a redistribution of inter-monomer interactions upon ligand binding, reflecting structural adaptation within the ectodomain. We also observed from the results that across all states, ssDNA was primarily bound to one monomer but stabilized by interactions from both.

For the TLR9_CpG_IM complex, we analyzed metastable states 1, 2, and 3 (Figure 8B). All CpG-TLR9 interactions observed in the TLR9_CpG complex were retained, and residues H612, H641, and K690 continued to interact with CpG bound to TLR9∗. Thus, CpG ssDNA remained stabilized by both monomers even after IM binding.

In the modeled complex, the IM adopted an orientation in which its 5′ end inserted into the TLR9 dimer while the 3′ end extended outward. RMSD analysis supported the stability of this configuration (Figure 2B). IM actively interacted with R348, S350, and R426 of TLR9 and interestingly, residues Y536, G565, and N567 of TLR9 contacted with the C and T nucleotides of IM bound to TLR9∗. These shared interactions between CpG, IM, and both monomers support cooperative binding and are consistent with the 2:2 stoichiometry of the TLR9_CpG complex and the 2:2:2 stoichiometry of the TLR9_CpG_IM complex observed previously.^24^ In TLR9_apo, residues R348-D534∗/Y536∗/G565∗/H566∗, F402-M561∗, and R426-S509∗/D534∗ of TLR9 formed pi-cation interactions, electrostatic interactions, or hydrogen bonds, but onlyR348-D534∗/Y536∗/G565∗/H566∗ and R426-D534∗ were observed in CpG bound state. Upon IM binding, however, R348, F402, and R426 re-engaged through pi-pi, electrostatic, and hydrogen-bond interactions with the 2C nucleotide of IM, consistent with interactions previously reported in mouse TLR9.^24^ These results suggest that ligand binding induces a coordinated redistribution of inter-monomer interactions and uncovers previously unrecognized functional residues (H612, H641, T642, and K690) that participate in stabilizing CpG and IM ligands. Based on our simulations, we hypothesize that CpG binding induces a conformational rearrangement around the Z loop that enables IM engagement, and that the orientation of IM further promotes favorable interactions between the 5′-xCx motif and the TLR9 dimer.

### Residue betweenness centrality analysis of apo- and CpG ssDNA and IM-bound TLR9 dimer complexes

**Figure 9:**
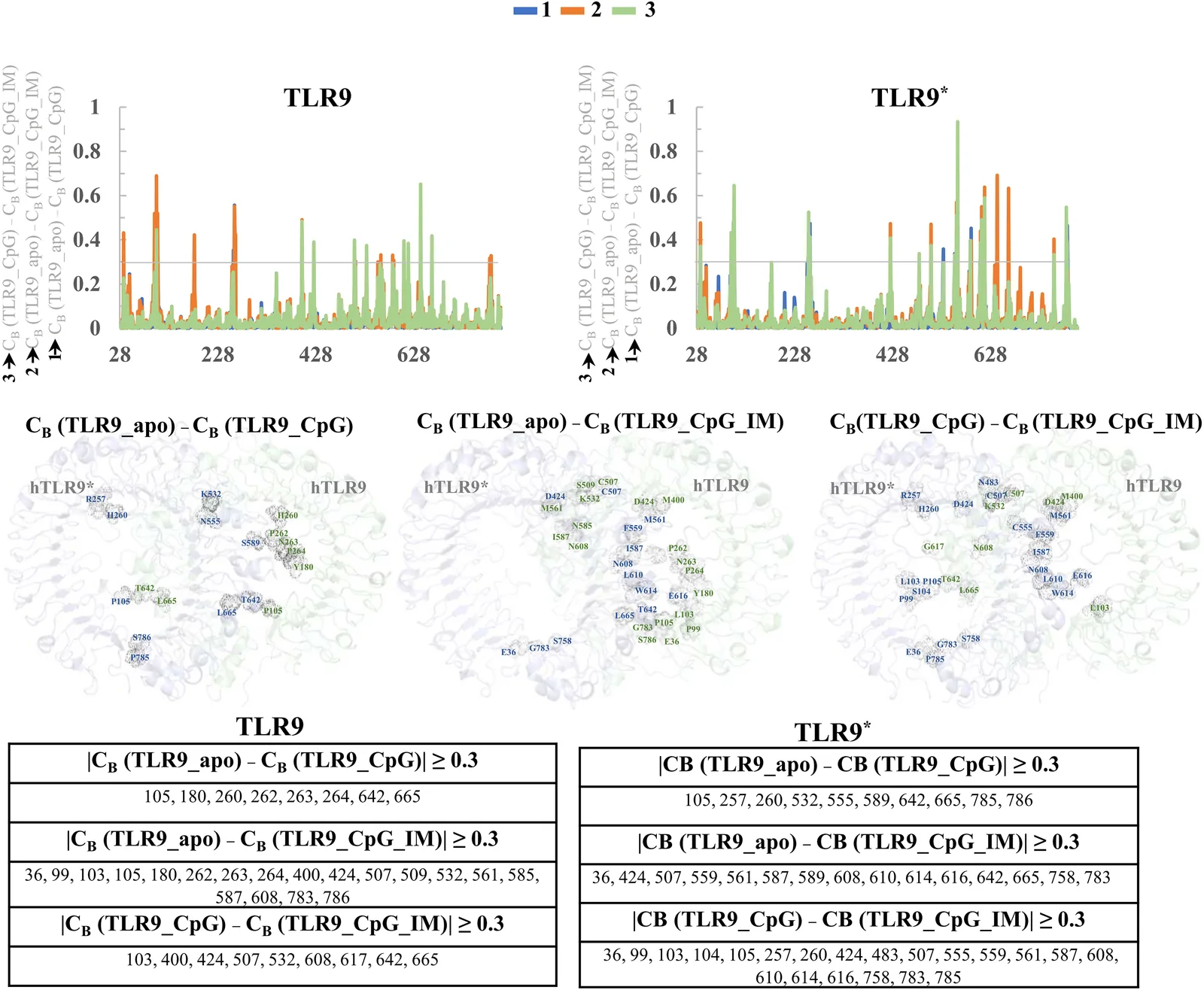
Residue C_B_ variations calculated between apo and ligand bound states of TLR9 The plot shows the difference in C_B_ among the three complexes with cutoff ≥0.3 (absolute values) for both monomers of TLR9. Locations of the residues observed ≥0.3 are mapped using dot representation on the structures and also listed on the tables.

To determine the critical residues in TLR9 and its ligand-bound complexes, we analyzed the betweenness centrality (C_B_) of these residues in the protein, which reflects the contribution of each node to intra-protein signal flow (Figure S4). Subsequently, to quantify changes in signal propagation between apo and ligand-bound states, we calculated the absolute differences |C_B_ (TLR9_apo) ^_^ C_B_ (TLR9_CpG) |, |C_B_ (TLR9_apo) ^_^ C_B_ (TLR9_CpG_IM) |, |C_B_ (TLR9_CpG) ^_^ C_B_ (TLR9_CpG_IM) | using a cutoff of ≥0.3 to identify residues with the largest shifts. These residues represent key allosteric nodes and are shown in Figure 9.

We examined the residues shared by the TLR9 and TLR9∗ monomers, and also reported residues in the dimer interface shared by TLR9∗ as they showed high CB variation. For |C_B_(TLR9_apo) – C_B_(TLR9_CpG)|, residues P105 (LRR2), H260 (LRR8), T642 (LRR21) and L665 (LRR22). Y180∗ (LRR5), K532∗ (LRR17), N555∗ (LRR18), S589∗ (LRR19), T642∗ (LRR21), L665∗ (LRR22), P785∗ (C-terminal LRR), and S786∗ (C-terminal LRR) exceeded the cutoff in TLR9∗ monomer. Among these, Y180 and T642 lie at the dimer interface and mediate inter-monomer interactions as seen in interaction analysis.

In contrast, for |C_B_(TLR9_apo) – C_B_(TLR9_CpG_IM)|, residues N-terminal LRR E36, D424 (LRR14), C507 (LRR16), M561 (LRR18), I587 (LRR19), N608 (LRR20) and G783(C-terminal LRR) were common residues in both monomers with high C_B_ values. C507∗ (LRR16), F559∗ (LRR18), M561∗ (LRR18), T642∗ (LRR21), and K738∗ (LRR25) showed the largest C_B_ differences in TLR9∗. Similar to the CpG-bound comparison, these residues cluster near the dimer interface and the Z loop, suggesting a role in stabilizing ligand-dependent conformational rearrangements. Although D424∗ (LRR14), C507∗ (LRR16), and F559∗ (LRR18) displayed high C_B_ values, they did not form direct interactions; however, their side chains project toward the interface region. Additional residues: I587, N608, L610, and W614 (LRR19–20), also exceeded the cutoff but are located away from the interface and do not orient toward the opposing monomer.

Comparison of |C_B_(TLR9_CpG) – C_B_(TLR9_CpG_IM)| revealed further shifts in the Z loop and LRRs 15–21, indicating altered communication pathways upon IM binding. All residues with C_B_ ≥ 0.3 for TLR9 monomers are listed in Figure 9. Notably, residues C507 and T642 have not been previously implicated in TLR9 function. In our analysis, however, both exhibit substantial increases in C_B_ upon ligand binding, suggesting that they may serve as previously unrecognized allosteric mediators linking the CpG and IM binding sites. Their pronounced network-level response indicates a potential role in long-range signal propagation within the receptor. Together, these shifts in C_B_ reveal a coordinated network of allosteric nodes spanning the dimer interface and Z-loop region, defining the pathways through which ligand binding reshapes signal propagation in TLR9.

## Discussion

TLR9 recognizes bacterial and viral CpG DNA and initiates innate immune signaling. In this study, we focus specifically on ectodomain-level structural rearrangements associated with ligand recognition. These mechanistic features cannot be inferred from static structures alone and emerge only when the human TLR9 ectodomain is examined dynamically, highlighting the value of a simulation-based approach. Because our analysis relies on a homology model of human TLR9, some structural uncertainty is unavoidable. However, the unusually high sequence identity and conserved LRR architecture shared with mice and equine TLR9 substantially reduce this uncertainty. Accordingly, our conclusions emphasize relative conformational changes and interaction patterns, which are robust across independent simulations, rather than absolute atomic positions.

TLR9 is highly conserved in its interior, non-exposed residues, which likely contribute to overall structural stability. In contrast, residues at the dimer interface show lower conservation. Several cross-monomer interaction residues identified in our simulations such as H612, H641, and K690 are not conserved in mouse or equine TLR9 and therefore cannot be inferred from existing crystal structures. This underscores the need for human-specific modeling. The 70%–80% sequence identity between human TLR9 and its orthologs enabled reliable homology modeling, and the modeled TLR9_apo structure remained stably dimeric throughout the simulations. This behavior is consistent with mouse and equine TLR9, which also exist as prefusion dimers.^21^^,^^24^ DCCM analysis further supported this prefusion arrangement, revealing positive correlations between the N terminuses of the monomers. Although the apo dimer is stable, it exhibits substantial global motions, as reflected in both DCCM and PCA analyses. Previous structural and biochemical studies have shown that Z-loop cleavage is required for TLR9 activation^21^ and our simulations are consistent with this model. In the apo state, the Z loop remains solvent-exposed and flexible, with residues in LRRs 15–23 contributing to a stable dimer interface. These conserved LRR regions form a robust interaction surface dominated by positively charged and hydrophobic residues, supporting prior observations that electrostatic and hydrophobic complementarity stabilize TLR9 dimerization. Although the apo dimer is stable, DCCM and PCA analyses reveal substantial global motions, indicating that the ectodomain samples multiple conformational substates that may prime the receptor for ligand engagement.

When CpG ssDNA from organisms like viruses or bacteria binds to TLR9, the Z loop in the TLR9_CpG complex fluctuates approximately 2-fold more than in TLR9_apo, suggesting increased accessibility for proteolytic cleavage. CpG binding also induces downstream conformational changes: LRR18 and LRR19 become negatively correlated with the N-terminal regions of the monomers, while the N-terminal regions of TLR9/TLR9∗ remain positively correlated. This pattern suggests that LRR18 and LRR19 move apart to accommodate IM binding. Several residues in LRR19–20 (I587, N608, L610, W614) exhibit high C_B_ despite lacking direct interactions, indicating potential functional relevance. Additionally, interactions present in the apo dimer (e.g., H641 and T642) are reassigned to the ssDNA ligands. CpG engages both the N-terminal LRRs of one monomer and the C-terminal LRRs of the opposing monomer, which likely contributes to the reduced anti-correlated motion observed between residues 100–300 of TLR9 and 700–800 of TLR9. LRR22 of one monomer also interacts with ssDNA bound to the partner monomer, stabilizing the complex. Because the number of TLR9-TLR9 contacts remains similar to the apo state, we propose that dimer interactions are redistributed to accommodate ligand binding.

Our findings align with mutational studies identifying Q346, R348, and Q562 as functionally important residues in human TLR9. Pohar et al.^29^ demonstrated that substitutions at R348 and Q562 impair signaling, highlighting their roles in ligand recognition. These residues correspond to R346, K348, and K563 in mouse TLR9 and to H346, K348, and R562 in equine TLR9, where they contribute to species-specific DNA preferences, specifically the substitution of Q346 in human TLR9 for R346 in mouse TLR9.^24^ Structural studies of equine TLR9 bound to CpG and 5′-xCx DNA show that H346 and K348 contact the third and fourth nucleotides of the 5′-xCx motif, while R562 forms part of the base of the 5′-xCx binding pocket.^25^ R562 in equine TLR9 lies within the loop region of LRR18 and forms part of the base of the 5′-xCx DNA-binding pocket. The residue projects toward the luminal face of the opposing protomer in the TLR9 dimer rather than directly contacting the 5′-xCx DNA itself. This positioning suggests that R562 (or Q562 in humans) may contribute to recognizing the 3′ extension of the DNA bound at the CpG-DNA binding site. Consistent with this idea, alanine substitution of Y345, F375, R377, F402, D534, Y536, and G560 in human TLR9 markedly reduces NF-κB activation in response to stimulation with the CpG oligodeoxynucleotide ODN10104.^28^^,^^29^ These observations support the functional relevance of the corresponding human residues and reinforce the mechanistic interpretations derived from our simulations.

TLR7, which shares mechanistic similarities with TLR9, adopts open and closed conformations with intermonomer distances of 6.93 nm and 3.65 nm, respectively.^33^ In contrast, we observe a modest 0.45 nm reduction in intermonomer distance between TLR9_apo and TLR9_CpG_IM. This difference is substantially smaller than the 3.2 nm shift reported for TLR7, likely reflecting the limited timescales accessible to atomistic simulations, which may not capture large-scale rearrangements in macromolecular receptors. Nevertheless, we detect subtle dynamic changes consistent with experimental findings. Many of these motions appear intrinsic to the receptor, with ligand binding suppressing inherent fluctuations and increasing rigidity. Most interactions observed in TLR9_CpG are preserved in the TLR9_CpG_IM complex. We observe that the binding of CpG and IM shifts several dimer-stabilizing interactions toward the ligands, resulting in a more stable dimer than in the apo form. The reduced intermonomer distance further supports ligand-induced stabilization of dimers. Together, these results support a stepwise activation mechanism in which CpG binding increases Z-loop flexibility, redistributes intermonomer contacts, and exposes the IM binding pocket.

The DNA IM used in this study represents a synthetic modulatory sequence whose relevance is supported by extensive structural and functional work. Equine and mouse TLR9 structures reveal a conserved 2:2 receptor-ligand arrangement, and human TLR9 activation requires two additional CpG motifs, indicating cooperative engagement of two DNA elements i.e., human TLR9 requires both CpG and 5′-xCx motifs for activation, whereas mouse TLR9 responds to CpG alone.^26^^,^^27^^,^^29^ Consistent with this, our results show 5′-xCx DNA enhances TLR9 dimerization in the presence of CpG DNA. Previous studies have shown that short 5′-xCx sequences can potentiate activation^21^^,^^24^ and more recent work shows that short 5′-xCx DNA potentiates TLR9 activation when co-present with CpG DNA.^28^ In this context, the IM provides one of the two required CpG motifs, with the second supplied by the ssDNA ligand. The resulting 2:2:2 configuration observed in our simulations aligns with known synthetic TLR9 modulators and experimentally supported stoichiometries. IM binding is stabilized by LRRs flanking the Z loop in both monomers, consistent with the opening of LRR18–19 upon CpG binding. The reduced Z-loop dynamics in TLR9_CpG_IM suggest that increased Z-loop flexibility in TLR9_CpG facilitates IM engagement. Elevated C_B_ in LRR26 across apo and ligand-bound states indicates altered long-range communication within the ectodomain. Similarly, the Y180/K181 pair also shifts from weak inter-monomer contacts with E616∗ in the apo form to interactions that contribute to dimer-interface stabilization (Y180) and CpG stabilization (K181) in the ligand-bound states. Because our simulations are limited to the ectodomain, any linkage between these rearrangements and downstream signaling remains inferential and will require validation in full-length receptor systems.

While these findings highlight potential allosteric nodes within the human TLR9 ectodomain, they should be viewed as testable predictions rather than definitive mechanistic determinants. Experimental interrogation of these residues, particularly H612, H641, T642, and K690, will be essential for establishing their roles. Furthermore, mutations at H612 or K690 could weaken CpG stabilization despite their lack of contact in the apo structure and disruption of Y180–E616∗ or C507-mediated interface contacts could impair the CpG-induced rearrangement required for IM binding. Another testable hypothesis is the residues with large shifts in C_B_, such as T642 and K738, which are predicted to mediate long-range allosteric communication between CpG and IM binding sites. These predictions provide a framework for targeted mutagenesis studies in human TLR9. Overall, our results indicate that the TLR9 dimer is intrinsically stable and that LRRs flanking the Z loop play key roles in coordinating CpG ssDNA and IM binding.

### Limitations of the study

This study modeled human TLR9 in its apo, CpG ssDNA-bound, and IM-bound forms using a homology model based on highly conserved TLR9 orthologs. While the model is strongly supported by high sequence identity and structural similarity, homology modeling inherently introduces uncertainty. As a result, our conclusions focus on relative conformational changes and interaction patterns, rather than absolute structural predictions. The key interactions we observe are consistent with experimentally resolved mouse and equine TLR9 structures, but human-specific nuances may not be fully captured. Our simulations support a 2:2 stoichiometry for TLR9-CpG ssDNA and a stable 2:2:2 arrangement for TLR9-CpG-IM, consistent with available crystal structures. However, we did not explore alternative stoichiometries or ligand orientations, which is a limitation of this study. Similarly, the diverse sequence specificity of CpG and IM ligands and the opposite IM orientation were not examined here. The rearrangement of interactions between monomers and ligands highlights residues important for CpG and IM recognition and stabilization. These findings suggest a mechanism in which CpG binding relaxes the LRR region around the Z loop, enabling IM engagement and promoting ligand-induced dimer stabilization. Nonetheless, our simulations capture only ectodomain-level rearrangements; the full multi-step activation process of TLR9, including TIR-domain reorganization, remains unresolved. Finally, this study lacks direct experimental validation. While the observed interactions align with known structural and biochemical data from multiple species, future experimental work will be essential to confirm the mechanistic insights proposed here.

## Resource availability

### Lead contact

Requests for further information can be directed to and will be fulfilled by the lead contact, Donghyun Shin (sdh1214@gmail.com).

### Materials availability

We have not generated unique reagents from this study.

### Data and code availability

• Initial structures subjected to simulations and coordinates extracted from free energy landscape is available from Figshare database (https://doi.org/10.6084/m9.figshare.31643326).
• No original code reported for this study.
• Any additional information can be requested from the lead contact.

## Acknowledgments

This study was supported by the National Research Foundation of Korea (NRF) grant funded by the Korean Government (10.13039/501100014188MSIT) (no. 2022R1A2C4002510). This research was supported by the Science and Technology Project Opens the Future of the Region through an 10.13039/501100012633INNOPOLIS Foundation grant funded by the 10.13039/501100014188Ministry of Science and ICT of Korea (2022-DD-UP-0333). S.S. has received support from a Michael Smith Health Research Trainee fellowship, and a Mitacs Accelerate Fellowship. We thank Dr. Sthitaprajna Sahoo from Jeonbuk National University for his assistance in generating the initial figures for formal analysis.

## Author contributions

S.S.: writing – original draft, methodology, investigation, formal analysis, and conceptualization; V.G.: writing – review and editing, methodology, supervision, investigation, formal analysis, resources, and conceptualization. D.S.: writing – review and editing, supervision, funding acquisition, and resources.

## Declaration of interests

The authors declare no competing interests.

## STAR★Methods

### Key resources table

**Table:** 

REAGENT or RESOURCE	SOURCE	IDENTIFIER
**Software and algorithm**		

GROMACS (v5.1.4)	Abraham et al.^34^	https://www.gromacs.org/
SWISS-MODEL	Waterhouse et al.^35^	https://swissmodel.expasy.org/
NAPS webserver	Chakrabarty et al.^36^^,^^37^	http://bioinf.iiit.ac.in/NAPS/
PDB	PDB ID: 3WPC, 5ZLN	https://www.rcsb.org/
Uniprot	Q9NR96	https://www.uniprot.org/
Figshare database	Initial and representative structures extracted in this study	https://doi.org/10.6084/m9.figshare.31643326

### Method details

#### TLR9 complex preparation

Although a few structures of horse and mouse TLR9 are available, the structure of human TLR9 is not solved. Therefore, we first retrieved the sequence of TLR9 ectodomain (ECD) from UniProt database (Q9NR96). The sequence identity between horse TLR9 and human TLR9 ECD was 83.6%, whereas that of mouse TLR9 was 71.3%. Hence, we generated 3D models of TLR9 ECD based on the horse TLR9 structure (PDB ID: 3WPC) using SWISS-MODEL webserver^35^ as a homodimer (hereafter, TLR9 and TLR9∗ monomers) and this structure was considered TLR9_apo. The horse TLR9 structure is available in a complex with CpG DNA; however, no IM is present. Therefore, to construct both TLR9_CpG and TLR9_CpG_IM complexes, we used a mouse crystal structure (PDB ID: 5ZLN). The root-mean-square deviation (RMSD) between the TLR9_apo homodimer and 5ZLN is 0.55 Å (for 1316 Cα atoms). We superimposed the TLR9_apo homodimer on to the mouse TLR9 structure (PDB ID: 5ZLN) and extracted the CpG ssDNA (single stranded) (nucleotide sequence: 5-AGGCGTTTTT-3′) (2:2 stoichiometry).^21^^,^^24^ Moreover, to construct TLR9_CpG_IM, we superimposed the TLR9_CpG DNA complex onto the mouse structure and extracted a single stranded immunomodulator (IM) (5′-xCx nucleotide sequence: 5′-TCGC-3′) at 2:2:2 stoichiometry. Subsequently, the three complexes were analyzed and prepared for simulations. Previous reports have suggested that binding of CpG DNA and IM to TLR9 directly promotes dimerization and augments activation^24^ and therefore, two monomers of TLR9 were maintained as modeled. Although homology modeling introduces inherent uncertainty, the high sequence identity between human and horse TLR9 and the low RMSD relative to the mouse TLR9 structure support the structural reliability of the model for studying ectodomain-level dynamics.

#### Molecular dynamics simulations of apo-, CpG ssDNA-, and IM-bound TLR9 complexes

TLR9 is localized in the intracellular compartments of the endosomes after signaling, and an acidic pH (5–6.5) is crucial for its interaction with CpG ssDNA.^38^ Therefore, we analyzed and modified the protonation of charged residues at pH 5.5, particularly the histidine residues, using H++ webserver.^39^ Subsequently, all TLR9 complexes were subjected to molecular dynamics simulations using GROMACS 5.1.4^34^ with the AMBER ff99SB-ILDN force field for both proteins and DNA. Furthermore, TLR9 complexes were placed in a dodecahedral box containing TIP3P water molecules. The periodic boundary distance was maintained at 14 Å, and the sufficient counter ions were added to neutralize the system. Simulations were performed in three steps. First, the complexes were subjected to energy minimization with a maximum tolerance of 1000 kJ/mol using the steepest descent gradient algorithm. Subsequently, the system was equilibrated using NVT and NPT ensemble for 500 ps and 1 ns, respectively. Finally, production simulations were performed using a 2 fs time step, integrated with leap-frog algorithm, for 300 ns with three independent replicates (3 × 300 ns). The simulation parameters are similar to our previous studies.^40^ In brief, particle-mesh Ewald (grid spacing of 0.16 nm) was used for fast Fourier transform and applied for long-range electrostatics with 1.0 cutoff for short-range and van der Waals interactions. The hydrogen bonds were constrained using Lincs algorithm. Temperature (300 K) and pressure (0.1 bar) were applied using modified Berendsen thermostat (v-rescale) and Parrinello-Rahman barostat. Finally, coordinate data were saved every 2 ps for trajectory analysis. Most trajectory analyses were performed using built-in GROMACS tools, such as RMSD, root-mean-square fluctuation (RMSF), hydrogen bonds, and density distributions. To calculate the interaction energy between components during the simulations, we used rerun module integrated into GROMACS. The top principal components (PCs) were extracted using gmx covar and gmx anaeig from a concatenated trajectory comprising the last 250ns from each replicate. The gmx covar calculates and parameterizes the matrix, and the eigenvectors were analyzed using gmx anaeig. The free-energy landscapes (FELs) of the top PCs were determined using gmx Sham module. A summary of the simulation parameters is given in Table S1.

#### Residue interaction networks

We constructed a non-weighted residue-residue interaction network using the NAPS web server to gain deeper insight into the intramolecular signal transduction mechanisms of the protein.^37^ The analysis was carried out for the lowest energy structures of apo-, ssDNA- and IM-bound complexes of TLR9 dimer. In brief, each amino acid residue is represented as a node and the edges present the inter-residue contacts. The Cα-Cα distance cutoff was set to 7 Å with non-weighted edges.^41^ The influence of side chains was accounted for by defining two coarse grained centers per residue: one representing the Cα backbone and the other representing the side chain heavy atoms distant from the Cα atom. The betweenness centrality (C_B_), a parameter that quantifies signal propagation within the protein, was computed to identify key residues acting as central nodes.^36^^,^^42^ The absolute value was calculated as the difference between the per residue C_B_ values of apo and respective complex forms.
