# Supplementary material for: Insights into human TLR9 structural dynamics and recognition of pathogen-associated CpG DNA using molecular dynamics simulation
Source: iScience. 2026 Aug 12;29(8):117060. doi: 10.1016/j.isci.2026.117060 (PMC13498979; doi:10.1016/j.isci.2026.117060)
Supplement: Supplementary file 1 [file mmc1.pdf]

**iScience, Volume *29***

## Supplemental information

# Insights into human TLR9 structural dynamics and recognition of pathogen-associated CpG DNA using molecular dynamics simulation

**Santanu Sasidharan, Vijayakumar Gosu, and Donghyun Shin**

# Supplemental Information

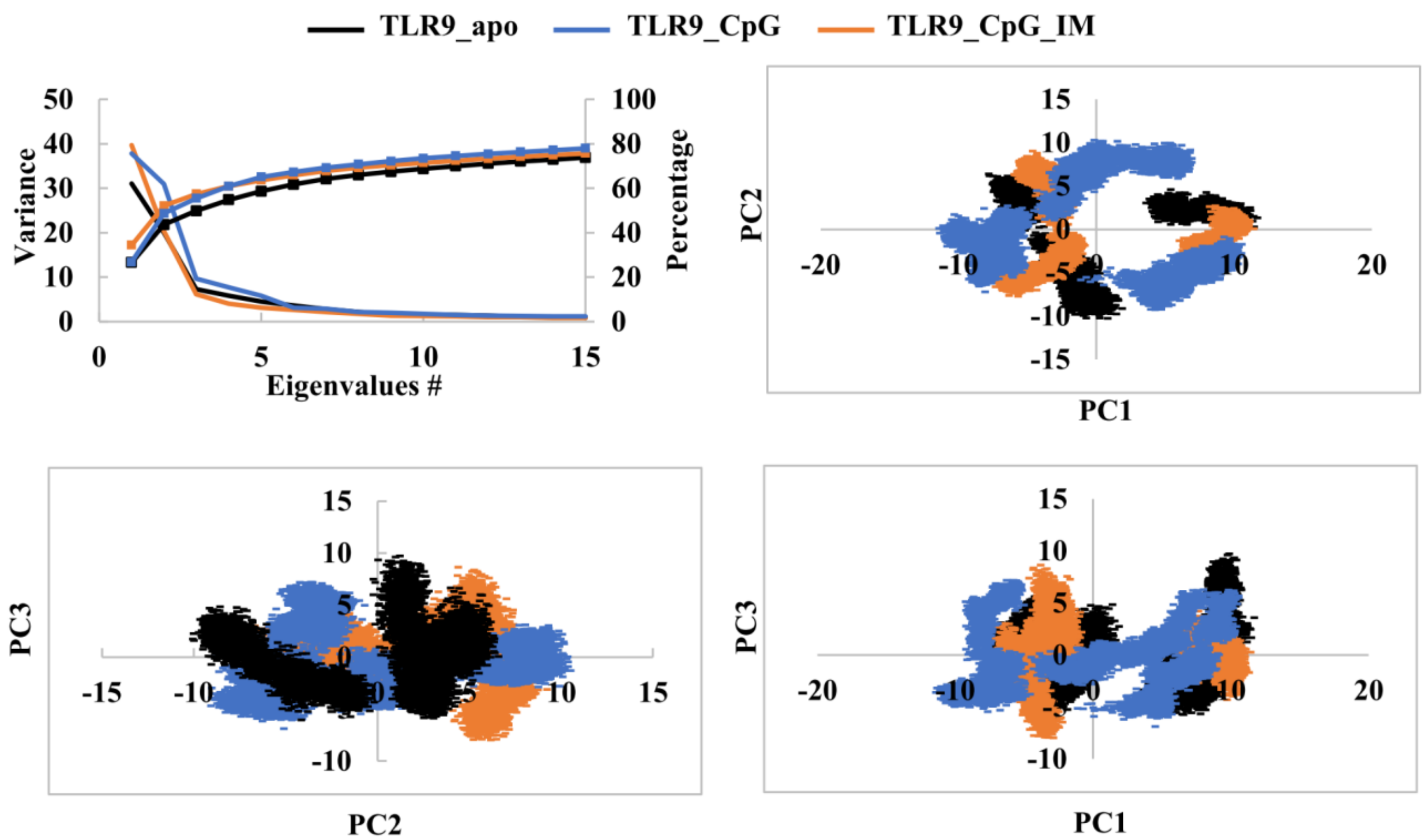


**Figure S1:** Principal component (PC) analysis. The eigenvalues and their cumulative variance are shown (A). Projections of PC1 vs. PC2 (B), PC2 vs. PC3 (C), and PC3 vs. PC1 (D) onto the phase space.

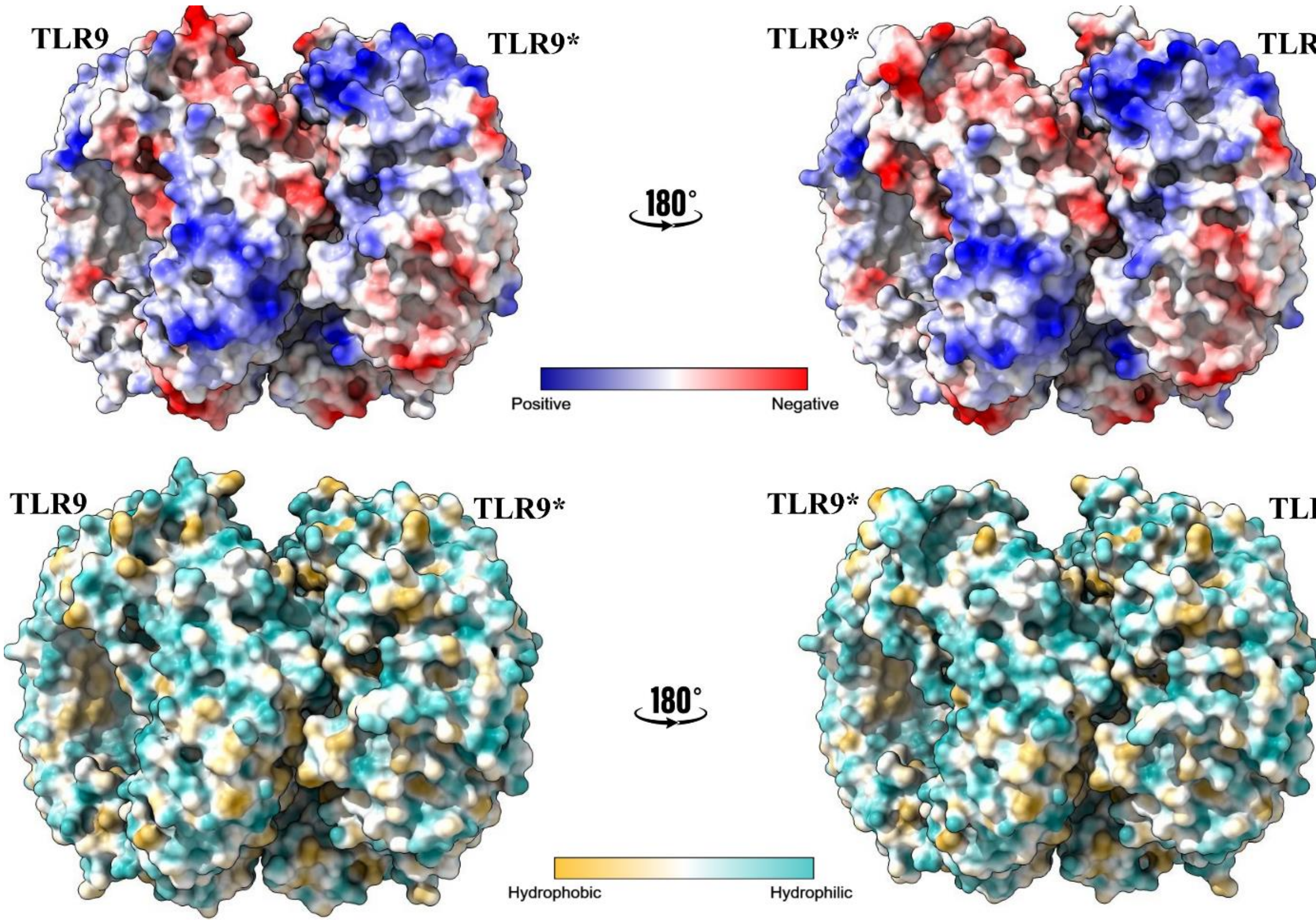


**Figure S2:** TLR9 dimer surface representation showing the distribution of charged residues at the dimer interface. The dimer interface has clusters of positively and negatively charged residues that contribute to the stabilization of the dimer in the apo form (Top). TLR9 dimer surface representation showing the distribution of hydrophobic and hydrophilic residues along the dimer interface (Bottom).

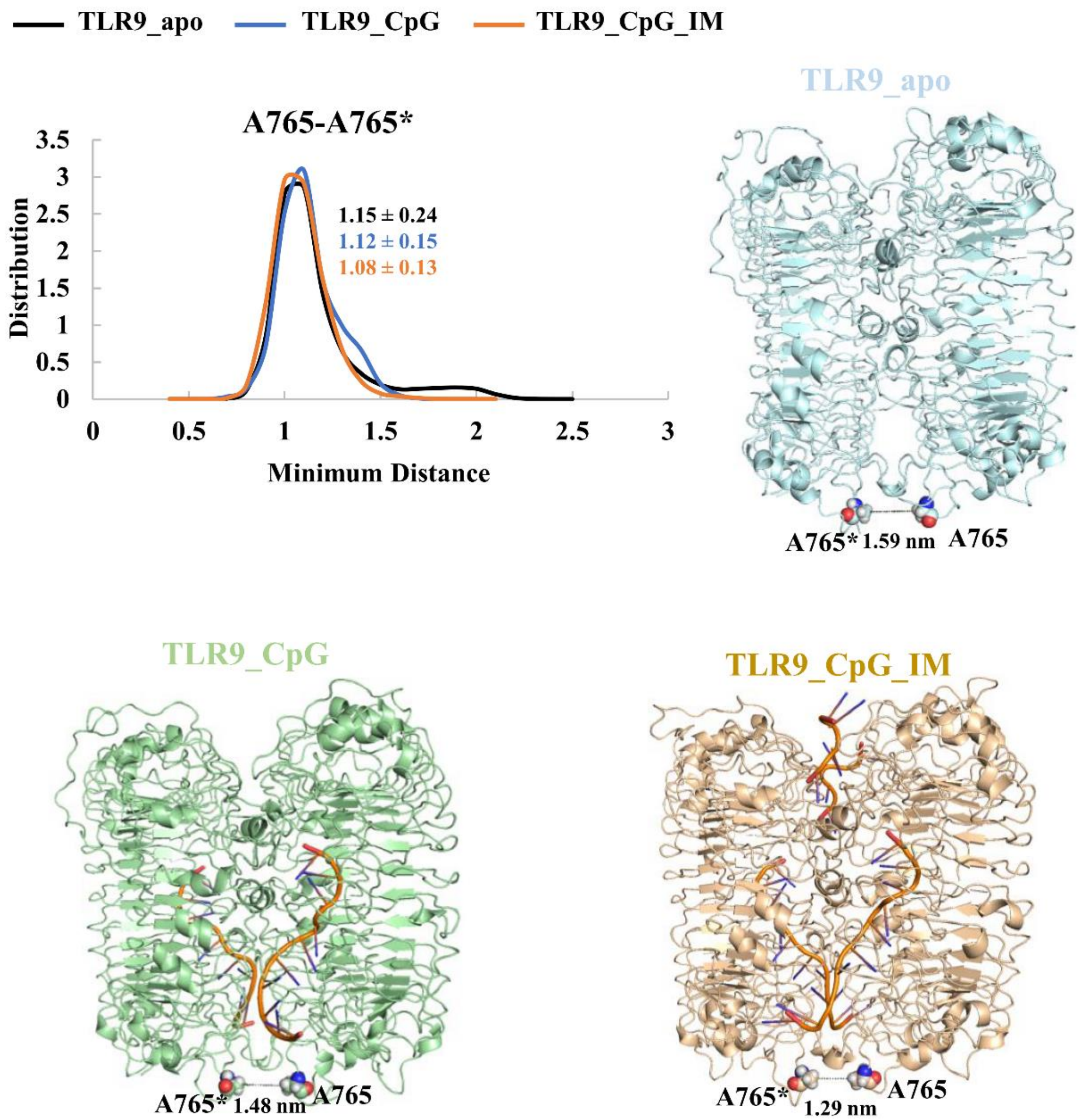


**Figure S3:** Distance analysis. Minimum distance between the corresponding residue (A765) from both monomers of Toll-like receptor 9 (TLR9) in all systems is shown. Location of the residue is indicated on the representative structures.

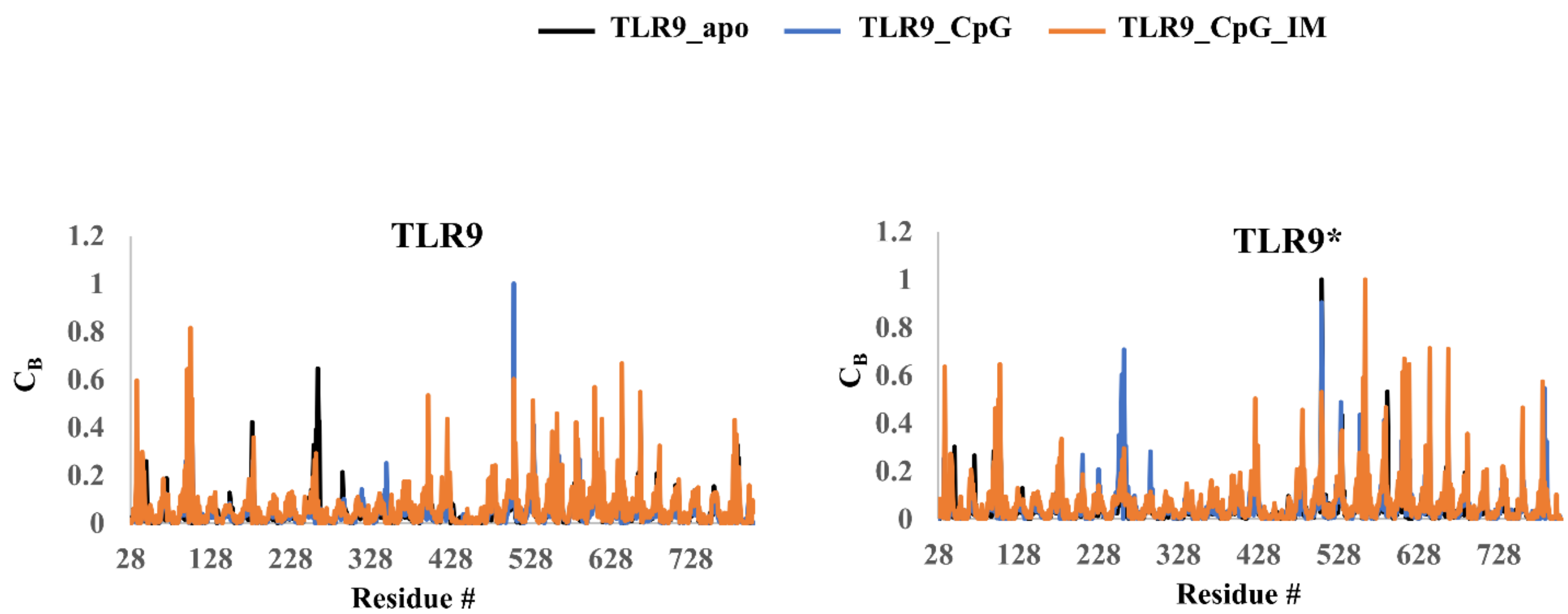


**Figure S4:** Residue betweenness centrality ($C_B$) calculated for the representative structures of all complexes.

**Table S1:** Molecular Dynamics Simulation Parameters for TLR9-CpG-IM Complexes

| Parameter | Value / Method |
|---|---|
| System size | ~196000 atoms for all the systems |
| Force field | AMBER ff99SB-ILDN (proteins and DNA) |
| Protonation state preparation | H++ server at pH 5.5 (histidines and other titratable residues adjusted) |
| MD engine | GROMACS 5.1.4 |
| Water model | TIP3P |
| Simulation box | Dodecahedral |
| Periodic boundary distance | 14 Å |
| Ions | Counter-ions added to neutralize the system |
| Energy minimization | Steepest descent; tolerance 1000 kJ/mol |
| Equilibration (NVT) | 500 ps |
| Equilibration (NPT) | 1 ns |
| Production simulation | 300 ns x 3 replicates |
| Time step | 2 fs |
| Integrator | Leap-frog |
| Electrostatics | Particle-mesh Ewald (PME), grid spacing 0.16 nm |
| Cutoffs | 1.0 nm for electrostatics and van der Waals |

| | |
|---|---|
| Constraints | LINCS (all bonds) |
| Thermostat | Modified Berendsen (v-rescale), 300 K |
| Barostat | Parrinello–Rahman, 0.1 bar |
| Trajectory output frequency | Every 2 ps |
